# Diagnostic and prognostic value of serum MACC1 in breast cancer patients

**DOI:** 10.18632/oncotarget.12910

**Published:** 2016-10-25

**Authors:** Weige Tan, Xinhua Xie, Laisheng Li, Hailin Tang, Xigang Ye, Lun Chen, Wei Tang, Jin Gao, Lingxiao Pan, Xiaoshen Zhang, Feng Ye, Xing Li, Lu Yang, Xiaoming Xie, Wenbo Zheng

**Affiliations:** ^1^ Department of Breast Surgery, the First Affiliated Hospital of Guangzhou Medical University, Guangzhou, 510120, China; ^2^ Department of Breast Oncology, Sun Yat-Sen University Cancer Center, Guangzhou, 510060, China; ^3^ Sun Yat-Sen University Cancer Center, State Key Laboratory of Oncology in South China, Collaborative Innovation Center for Cancer Medicine, Guangzhou, 510060, China; ^4^ Department of Laboratory Medicine, the First Affiliated Hospital of Sun Yat-Sen University, Guangzhou, 510080, China

**Keywords:** MACC1, breast cancer, diagnosis, biomarker, serum

## Abstract

Metastasis-associated in colon cancer-1 (MACC1) promotes colorectal cancer progression and predicts prognosis. The aim of our study was to determine the diagnostic and prognostic value of preoperative serum MACC1 levels in breast cancer patients. Serum MACC1 levels were measured in 378 breast cancer patients, 120 patients with benign breast disease, and 40 healthy volunteers using an ELISA. Serum MACC1 levels were higher in breast cancer patients than patients with benign disease or healthy volunteers. Increased serum MACC1 was associated with breast cancer TNM stage (*P* < 0.001), tumor size (*P* < 0.001), lymph node metastasis (*P* < 0.001), and Ki-67 status (*P* = 0.014). Serum MACC1 measurement successfully discriminated breast cancer patients from normal and healthy controls (AUC = 0.785, 95% CI: 0.746–0.825) with an optimal cut-off value of 38.35 pg/ml (sensitivity = 0.725, specificity = 0.696). Moreover, serum MACC1 exhibited significant prognostic value in breast cancer (AUC = 0.757, 95% CI: 0.700–0.814), and high MACC1 was associated with poor disease-free survival (HR 5.63, 95% CI: 3.51–9.04; *P* < 0.001). Our findings demonstrated that circulating MACC1 could serve as a reliable diagnostic and prognostic biomarker for breast cancer.

## INTRODUCTION

Breast cancer (BC) is the most frequently diagnosed cancer and the second leading cause of cancer-related death among American women [1, 2]. BC incidence has increased in China in recent decades and outcomes for patients with metastatic disease remain poor, with a median overall survival time of two to three years [3, 4]. A lack of effective treatment options, which rely heavily on timely diagnosis, contributes to poor survival in early-stage BC patients [5]. Novel biomarkers are urgently needed to detect early stage BC. However, many identified biomarkers [6–8], such as cancer antigen-199 (CA199), carcinoembryonic antigen (CEA), and cancer antigen-125 (CA125), have little clinical value due to low sensitivity, specificity, and reproducibility. Still, serum RNAs and proteins found to correlate with tumor status and/or patient survival are increasingly being applied as diagnostic and prognostic indicators in various carcinomas. Thus, detection of circulating proteins represents a promising noninvasive strategy for tumor diagnosis and prognosis, and for monitoring antitumor therapies.

Metastasis-associated in colon cancer-1 (MACC1), a newly identified gene first detected in colorectal cancer, is suggested to transcriptionally regulate c-Met [9]. MACC1 promotes human gastric cancer cell proliferation and invasion [10–12], and is overexpressed in diverse human malignancies, including BC [13–16]. A previous study associated MACC1 polymorphisms with HER2-positive BC patient clinical outcome, suggesting that MACC1 is a potential BC biomarker [17]. However, the association between BC and serum MACC1 levels has not yet been investigated.

Stable, repeatable, noninvasive molecular marker measurements could improve diagnostic and prognostic accuracy in cancer patients, and enable improved treatment decision-making [18, 19]. Based on previous findings, we hypothesized that serum MACC1 levels hold diagnostic and prognostic value in BC. In the current study we retrospectively examined serum MACC1 status in BC patients, patients with benign breast tumors, and healthy volunteers to assess the value of MACC1 as a biomarker.

## RESULTS

### Patient characteristics

This study included 378 breast cancer patients, 120 patients with benign breast tumors and 40 normal healthy controls. Study subject demographic, pathologic, and clinical information is provided in Table 1. Patient median age was 48.3 years. Eighty-one (21.4%) BC patients developed loco-regional or distant recurrence during follow-up. With a median follow-up of 69.45 months (ranging from 7.3 to 120.4 months), 5- and 10-year disease-free survival (DFS) rates were 80.4% and 77.6%, respectively. Clinically, 267 (70.6%) patients had a large tumor size (> 2 cm), and 211 (55.8%) patients had positive axillary lymph nodes. Most tumors were ER-positive (316/378, 83.6%), PR-positive (325/378, 86.0%), and HER2-negative (332/378, 87.8%), with Ki-67 ≥ 14% (223/378, 59.0%). Two hundred ninety-one patients (77.0%) received an anthracycline- or taxanes-based regiment. Forty patients (10.6%) received breast-conserving surgery; 338 patients (89.4%) received a modified radical mastectomy (MRM).

**Table 1:** Clinicopathological variables of breast cancer patients and controls

Variables		Breasr cancer patients (*n* = 378)	Breast benign tumors (*n* = 120)	Healthy (*n* = 40)
Age	≤ 40 years	58 (15.3%)	76 (63.3%)	12 (30%)
	> 40 years	320 (84.7%)	44 (36.7%)	28 (70%)
Menopause	Yes	238 (63.0%)	32 (26.7%)	
	No	140 (37.0%)	88 (73.3%)	
Tumor size	≤ 2cm	111 (29.4%)	67 (55.8%)	
	> 2cm	267 (70.6%)	53 (44.2%)	
LN status	Negative	167 (44.2%)		
	Positive	211 (55.8%)		
TNM stage	I	82 (21.7%)		
	II	188 (49.7%)		
	III	108 (28.6%)		
Tumor Grade	I	37 (9.8%)		
	II-III	341 (90.2%)		
ER	Negative	62 (16.4%)		
	Positive	316 (83.6%)		
PR	Negative	53 (14.0%)		
	Positive	325 (86.0%)		
HER2	Negative	332 (87.8%)		
	Positive	46 (12.2%)		
Ki67	≤ 14%	155 (41.0%)		
	> 14%	223 (59.0%)		
Surgery	Mastectomy	279 (73.8%)		
	BCS	99 (26.2%)		
Chemotherapy	No	87 (23.0%)		
	Yes	291 (77.0%)		

### Association between serum MACC1 levels and clinicopathological variables

**Figure 1:**
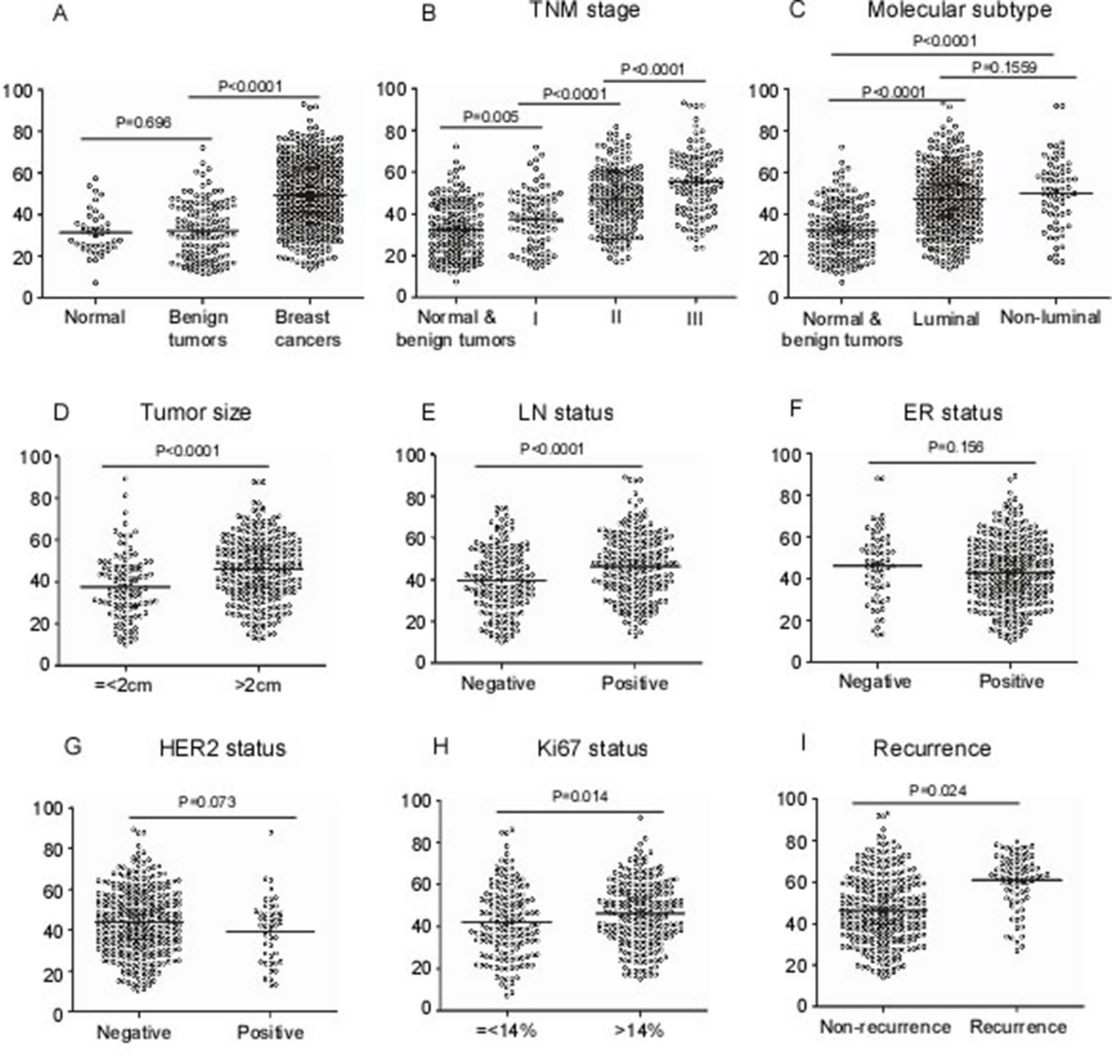
Association between serum MACC1 levels and clinicopathological variables Comparison of serum MACC1 levels between benign tumor and healthy controls in BC patients (**A**); in healthy and benign controls and BC patients at different TNM stage (**B**); in BC patients with different molecular subtypes (**C**); in BC patients with different tumor sizes (**D**); in LN-positive and LN-negative BC patients (**E**); in ER-positive and ER-negative breast cancer patients (**F**); in HER2-positive and HER2-negative breast cancer patients (**G**); in breast cancer patients with Ki-67 ≤ 14% and > 14% (**H**); in breast cancer patients with and without local/distant recurrence (**I**).

We measured serum MACC1 levels in BC patients and normal healthy controls by ELISA. Mean serum MACC1 was elevated in BC patients (53.43 ± 15.89 pg/mL) compared with healthy controls (38.22 ± 12.93 pg/mL) (*P* < 0.0001, Figure 1A). Compared with the healthy control and benign tumor groups, serum MACC1 were elevated in BC patients at any TNM stage (I, II or III) (Figure 1B). This trend was also evident in patients with tumor size > 2 cm (Figure 1D). Furthermore, serum MACC1 levels were higher in patients with lymph node metastases compared to those without lymph node metastases (56.34 ± 15.53 pg/mL, 49.74 ± 15.60 pg/mL, respectively; *P* < 0.0001) (Figure 1E). Ki-67 expression in tumor tissues was consistent with serum MACC1 (Figure 1H). However, MACC1 level was not correlated with ER or Her2 status (Figure 1C, 1F and 1G), or presence or absence of distant metastases (56.97 ± 15.16 pg/ mL, 52.46 ± 16.78 pg/mL, respectively; *P* = 0.024) (Figure 1I).

### Diagnostic value of serum MACC1 in BC patients

**Figure 2:**
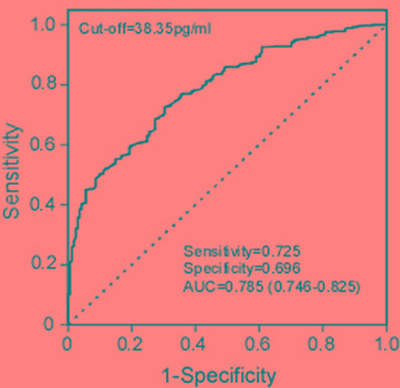
ROC analyses for serum MACC1 to differentiate breast cancers from healthy and benign tumor controls

To evaluate serum MACC1 as a BC diagnostic biomarker, we calculated the ROC by plotting sensitivity against specificity for serum MACC1 in different groups. We found that serum MACC1 successfully discriminated BC patients from healthy controls (AUC = 0.785, 95% CI: 0.746–0.825). An optimal cut-off value (38.35 pg/ml), which is of critical importance to accurate BC diagnosis, was determined by the score closest to the value under peak sensitivity (0.725) and specificity (0.696) (Figure 2).

### Serum MACC1 levels predict DFS of BC patients

**Figure 3:**
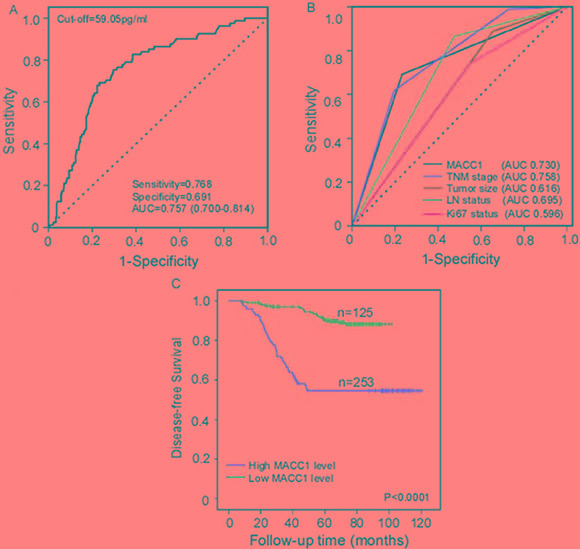
Serum MACC1 levels predict BC patient DFS ROC analyses for serum MACC1 to predict BC patient DFS. (**A**) Comparison of different clinicopathological factors in predicting BC patient DFS (**B)**. BC patient Kaplan-Meier survival curves (**C**). DFS rates of BC patients with high (> 59.05 pg/ml) and low (≤ 59.05 pg/ml) serum MACC1 levels.

Serum MACC1 levels were effective BC prognostic indicators as shown by ROC analysis (AUC = 0.757, 95% CI: 0.700–0.814). An optimal cut-off value (59.05 pg/ml) was determined by the score closest to the value under peak sensitivity (0.768) and specificity (0.691), as a threshold to partition the 378 BC patients into two groups: high serum MACC1 (MACC1 > 59.05 pg/ml, *n* = 125) and low serum MACC1 (MACC1 ≤ 59.05 pg/ml, *n* = 253) (Figure 3A). We found that MACC1, like TNM stage, predicted BC patient DFS (AUC = 0.730, or AUC = 0.758; Figure 3B). As shown by Kaplan-Meier log rank analysis, higher serum MACC1 levels (median survival time, 40.5 months) correlated with poorer DFS compared with lower serum MACC1 levels (median survival time, 67.2 months) (HR 5.63, 95% CI: 3.51–9.04; *P* < 0.001; Figure 3C). Table 2 summarizes the value of various risk factors in predicting BC patient prognosis, using univariate and multivariate Cox analysis.

**Table 2:** Univariate and multivariate Cox analysis of variables considered for disease-free survival rates of breast cancer patients

Variables	Category	Univariate			Multivariate		
		HR	95% CI	*P* value	HR	95% CI	*P* value
Age	> 40 vs. ≤ 40 years	0.64	0.37–1.09	0.098	0.68	0.38–1.19	0.174
Tumor size	> 2 vs. ≤ 2cm	3.53	1.77–7.06	0.000	1.48	0.71–3.06	0.295
LN status	Positive vs. Negative	5.65	2.99–10.67	0.000	3.11	1.60–6.02	0.001
TNM stage	Stage II-III vs.I	24.68	3.43–177.39	0.001	4.53	0.56–36.51	0.156
Grade	Grade II-III vs.I	3.00	0.95–9.50	0.062	2.04	0.63–6.68	0.237
ER status	Positive vs. Negative	0.96	0.54–1.72	0.903	1.45	0.79–2.65	0.226
HER2 status	Positive vs. Negative	1.14	0.59–2.22	0.693	1.45	0.72–2.92	0.301
Ki67 status	> 14% vs. ≤ 14%	2.16	1.31–3.54	0.002	2.23	1.34–3.69	0.002
MACC1	> 59.05 vs. ≤ 59.05 pg/ml	5.63	3.51–9.04	< 0.0001	4.91	3.03–7.95	< 0.0001

## DISCUSSION

Circulating biomarkers are regarded as reliable indicators in the diagnosis, monitoring and prognosis of numerous cancers [20–23]. Many serum biomarkers are useful in diagnosing BC [8, 24], with CA153 and CA125 most widely applied. However, low sensitivities and specificities limit the clinical application of such markers, especially for early BC diagnosis. In the current study, the diagnostic and prognostic values of serum MACC1 in BC patients were investigated and evaluated. Serum MACC1 levels were elevated in BC patients compared with patients with benign breast diseases or healthy volunteers. Our ROC analysis results suggest that serum MACC1 can distinguish BC patients from healthy controls, with a sensitivity of 71.4%, specificity of 89.1%, and AUC of 0.766. We therefore conclude that serum MACC1 as a biomarker can assist clinicians in diagnosing BC.

Our data showed that serum MACC1 levels were associated with clinical TNM stage, tumor size, lymph node status and Ki-67 status, but not ER and HER2 status, which are universally acknowledged as important BC prognostic biomarkers. We hypothesized that serum MACC1 levels may mirror BC tumor progression and invasion. Consistent with our findings, a separate study associated increased MACC1 in tumor tissues with progressive factors, such as Ki-67 status, TNM stage, tumor size, and lymph node status [25, 26], excluding ER and HER2 status [17]. The association between serum MACC1 and clinical TNM staging in the current study may be explained by the fact that most serum MACC1 originates from tumor tissues. Further research is needed to determine whether or not MACC1 directly contributes to BC development or progression [9, 27].

Kaplan-Meier and Cox regression analyses revealed that high serum MACC1 level was correlated with poor DFS and could be an independent prognostic factor for BC. While a previous study demonstrated that MACC1 mRNA polymorphisms were associated with HER2-positive BC patient clinical outcome, our study provides the first evidence that serum MACC1 may be an optimal diagnostic and prognostic BC biomarker. Serum MACC1 can be easily tested in clinical laboratories using a commercially available kit.

However, our study had several limitations. First, our study involved a relatively small number of patients, and larger multicenter studies are needed to confirm our results. Second, MACC1 expression in serum is not specific for BC. Others cancers, such as lung and colorectal cancers, exhibit high MACC1 serum levels and may thus impair accurate BC diagnosis [28, 29]. Third, CA153, CEA and CA125 were not detected in control groups, so the diagnostic power of serum MACC1 cannot be compared with existing BC biomarkers. In addition, serum samples in this study came from two hospitals and study results may differ due to system error. However, we excluded all known confounding factors from this study. In the future, molecular studies should evaluate potential roles for MACC1 in promoting BC, and large, prospective cohort studies should evaluate serum MACC1 as a marker for screening response to neoadjuvant chemotherapy.

In conclusion, our study demonstrated that serum MACC1 levels were elevated in BC patients compared with control groups, suggesting that MACC1 might act as a useful serum biomarker for distinguishing between early BC patients and non-BC controls. Additionally, BC patients with higher serum MACC1 levels had poorer survival, indicating that blood MACC1 levels might serve as a prognostic biomarker.

## MATERIALS AND METHODS

### Patients

Three hundred and seventy eight stage 0–III breast cancer patients undergoing mastectomy or breast conserving surgery, diagnosed and treated from January 2005 to January 2008 in Sun Yat-sen University Cancer Center, were enrolled in this study. All invasive BC patient diagnoses were confirmed independently by two pathologists who reviewed pathological slides from biopsies or resected tissues. Patients who received neo-adjuvant chemotherapy before surgery were excluded from this study. All BC patients received standard treatment with routine therapy, chemotherapy, and radiotherapy after surgery according to National Comprehensive Cancer Network guidelines. All patient histopathological classification was determined according to World Health Organization criteria, and staged classification was defined according to the Union for International Cancer Control TNM staging system. The control group included 120 patients with benign breast diseases such as phyllode tumor of the breast, breast intraductal papilloma, and mammary fibroadenoma, and 40 healthy volunteers from the First Affiliated Hospital of Sun Yat-Sen University. None of the control patients had previously been diagnosed with any malignancy. Blood samples were taken from patients on the day of diagnosis, prior to any surgery or therapy. All patients were followed up every three months by telephone or correspondence. Clinical assessments, including routine physical examinations, blood tests, breast and lymph ultrasonography, bone scintigraphy, or imaging studies, were performed for all patients every 3–6 months. The study was completed on August 30, 2016. The study end point was locoregional or distant recurrence of disease. For patients who underwent surgery, DFS was defined as the period from diagnosis to first locoregional or distant recurrence.

The study protocol was approved by the independent ethical committee/institutional review board of Sun Yat-sen University and Sun Yat-sen University Cancer Center. Written informed consent regarding the scientific research was obtained from each participant prior to surgery. Patient records were anonymized and de-identified prior to analysis.

### Serum preparation and MACC1 detection

Serum samples, which were collected at the time of cancer diagnosis and stored at -80°C, were obtained from the department of breast oncology in our cancer center.. A double-antibody sandwich ELISA was conducted to detect serum MACC1 using an ARCHITECT i2000 SR system (Abbott Laboratories, Chicago, USA) according to the manufacturer's protocol. The MACC1 ELISA kit was purchased from R&D Systems (Minneapolis, MN, USA).

### Statistical analyses

Pearson chi-square test or Fisher's exact test was used to assess categorical values. Mann-Whitney U test and Kruskal-Wallis test were used to determine differences between groups. Mann-Whitney *U* test or the Wilcoxon matched pairs test was applied to evaluate associations between MACC1 levels and various BC clinicopathological variables. Receiver operating characteristics (ROC) analysis was applied to determine serum MACC1 sensitivity and specificity in discriminating between breast cancer, benign tumors and healthy controls or stratifying patients by recurrence risk. Area under the ROC curve (AUC), sensitivity and specificity were used to assess the diagnostic power of serum MACC1. The cut-off value was determined by the score closest to the value under peak sensitivity and specificity. Survival rates and curves were determined by the Kaplan-Meier method, and differences in survival were evaluated using the log-rank test. COX regression analysis was used for univariate and multivariate analysis of correlation between clinicopathological variables and overall survival. All statistical analyses were performed using Graphpad Prism version 5.0 (GraphPad Software Inc., San Diego, CA, USA) and SPSS version 13.0 (SPSS Inc., Chicago, IL, USA). *P* < 0.05 was considered statistically significant, and all statistical tests were two-sided.
